# Knowledge, Health Beliefs, and Self-Efficacy regarding Osteoporosis in Perimenopausal Women

**DOI:** 10.1155/2013/853531

**Published:** 2013-09-11

**Authors:** Renée D. Endicott

**Affiliations:** University of Missouri-Kansas City, School of Nursing and Health Sciences, 2464 Charlotte Street, Kansas City, MO 64108-2639, USA

## Abstract

The aims of this pilot study were to (1) determine if having a family history of osteoporosis impacts knowledge, health beliefs, and self-efficacy regarding osteoporosis among perimenopausal women aged 42–52 and to (2) describe the impact of an osteoporosis-specific educational intervention had on the knowledge, health beliefs, and self-efficacy of this population. Participants completed three surveys measuring knowledge, health beliefs, and self-efficacy related to osteoporosis before and two months after the educational program. At baseline, no differences were noted in knowledge of osteoporosis among women with and without a family history of osteoporosis, although women with a family history perceived a greater susceptibility for developing osteoporosis than women without the family history. Findings indicate that both groups increased in knowledge of osteoporosis (*P* < .001). Benefits of calcium increased in the women without a family history of osteoporosis (*P* < .001) and benefits of exercise increase in women with a family history of osteoporosis (*P* = .007). There were no significant statistical findings regarding self-efficacy between the two groups of women. Findings indicate that an osteoporosis-specific educational program improves perimenopausal women's knowledge and some health beliefs.

## 1. Introduction

Osteoporosis is a silent metabolic process that can potentially cause fracture, disability, and increased mortality [1–3]. Osteoporosis is a significant clinical and public health concern [3]. The National Osteoporosis Foundation (NOF) estimates that ten million individuals already have osteoporosis and almost 34 million have low bone mass, which places them at increased risk for developing osteoporosis [2]. There are a disproportionate number of women with the disease. Of the ten million Americans estimated to have osteoporosis, eight million are women [2]. A woman's risk of breaking her hip due to osteoporosis is equal to her risk of breast, ovarian, and uterine cancers combined [2].

There are many lifestyle risk factors for developing bone loss and osteoporosis such as a diet with poor calcium intake, lack of exercise, tobacco use, and alcohol intake [1, 2]. Additionally, genetics is a factor. Women 35 years and older with a family history of osteoporosis have almost twice the risk of developing the disease, compared to women without a family history [4]. The NOF estimated that 20% of non-Hispanic Caucasian and Asian women aged 50 and older have osteoporosis, and 52% are estimated to have low bone mass [2]. The NOF reports that one in every two Caucasian women will experience an osteoporotic fracture at some point in her lifetime [2]. 

Bone metabolism occurs throughout life. It involves repetitive turnover cycles of bone osteoclasts breaking down the bone structure, which is referred to as resorption and bone osteoblast building up the bone structure, a process known as bone remodeling [5]. In both sexes, peak bone mass is reached within three years after linear growth stops [6]. In women estrogen is needed to keep a healthy balance between bone resorption and bone remodeling [5]. Perimenopausal women are especially vulnerable to bone loss due to the fluctuating and declining estrogen levels. During the perimenopausal transition, serum estradiol levels can fall from 10% to 20%, and the level of serum estrone which is a fourfold weaker than estrogen falls to about 25% to 35% of the premenopausal level. During this time, bone resorption can increase by 90%, whereas bone formation increases by only 45% [5]. This imbalance in bone resorption and remodeling leads to accelerated bone loss [5]. In the first five to seven years after menopause, a woman can lose up to 20% of her bone density, and this loss can lead to osteoporosis [2]. 

The progression of bone loss that leads to osteoporosis can be slowed or delayed with lifestyle changes, particularly a diet with adequate calcium and vitamin D, weight-bearing physical exercise, smoking cessation, and reduction in alcohol use [1, 2]. Medications for osteoporosis are also available to minimize bone loss and prevent fracture [1, 2]. Previous studies have shown that many women of all ages lack knowledge about osteoporosis or do not perceive themselves as being at risk for developing bone loss and osteoporosis [7–11]. However, there is limited published literature specifically on perimenopausal women's knowledge, health beliefs, and self-efficacy regarding osteoporosis. It is important to examine the family history, knowledge, health beliefs, and self-efficacy of perimenopausal women and to provide them adequate education. Promoting bone health in this group may reduce morbidity later in life [12]_._

The aims of this pilot study were to (1) determine if having a family history of osteoporosis impacts knowledge, health beliefs, and self-efficacy regarding osteoporosis among perimenopausal women (between the ages of 42 and 52) compared to women without a family history of osteoporosis and (2) describe the impact an osteoporosis-specific educational intervention had on the knowledge, health beliefs, and self-efficacy for this same population. 

The theoretical model for this study was the Expanded Health Belief Model (EHBM). The Health Belief Model (HBM) is a conceptual framework used to understand health behaviors and reasons for noncompliance with recommendations for health actions [13, 14]. The Health Belief Model was developed in the 1950s by social psychologists working for the United States Public Health Service (USPHS) as a way to explain why medical screening programs were not successful [14–16]. The HBM has four major components for compliance with recommended health action: perceived barriers of recommended health action, perceived benefits of recommended health action, perceived susceptibility to the disease, and perceived severity of the disease [16]. Later the model was expanded to include cues to action and motivating factors [16]. In 1988, self-efficacy was added as a component. The core assumptions of the self-efficacy are based on the belief that three criteria primarily affect whether or not a person will take a health-related action. These three criteria are the conviction that one has the ability to initiate the activity, maintain the activity, and persist in performing the activity in the face of obstacles [17].

## 2. Methods

For this study, a case control design was used to compare the knowledge, health beliefs, and self-efficacy of women with a family history of osteoporosis and women without a family history of osteoporosis. A pre- and posttest design was used to examine whether knowledge, health beliefs, and self-efficacy regarding osteoporosis changed after being presented in the osteoporosis-specific educational program. Family history is defined as a first-degree relative and, or a grandparent. 

### 2.1. Participant Selection

This study received Institutional Review Board approval. Eligible participants were English speaking women between the ages of 42 and 52 still having menstrual cycles. Women were excluded if they had gone 12 or more months without a menstrual period, or had had both ovaries surgically removed, and/or they had previously been diagnosed with osteoporosis. Participants were recruited from an internal medicine practice with five suburban clinics. A report generated in the electronic medical record system identified women aged 42–52 years. Four hundred letters were sent to potential participants inviting them to participate in the study. Recruitment fliers were also placed in the waiting rooms of the clinics. Some of the women who participated in the study recommended the study to their friends, which was helpful in recruiting participants. Recruitment of the participants spanned a six-month period. 

### 2.2. Data Collection

 The participants consented to the study before participating. Baseline demographic information regarding age, race, educational level, marital status, and family history of osteoporosis was obtained from the participants. The participants then completed three valid and reliable standardized tools: *the Osteoporosis Knowledge Test* (OKT), *the Osteoporosis Health Belief Scale* (OHBS), and *The Osteoporosis Self-Efficacy Scale* (OSES). Permission to use these tools was granted by a coauthor of these instruments (P. Gendler, personal communication, October 27, 2011). Study materials were coded, which provided the ability to compare study materials, while maintaining confidentiality of the responses. A study code book was securely maintained by the researcher.

### 2.3. Osteoporosis Knowledge Test

 The OKT (revised 2011) is a 32-item multiple choice instrument that measures knowledge of osteoporosis. The OKT has two subscales: OKT Nutrition (items 1–11 and 18–32) and OKT Exercise (items 1–17 and 30–32). The OKT Nutrition and OKT Exercise subscales both share 14 common items (items 1–11 and 30–32). The OKT was developed by Katherine Kim, Ph.D., Mary Horan, Ph.D., and Phyllis Gendler, Ph.D. and was revised by Phyllis Gendler, Ph.D., Cynthia Coviak, Ph.D., Jean Martin, Ph.D., and Katherine Kim, Ph.D. Question 26 was developed as an addition to the Osteoporosis Knowledge Test by Pamela von Hurst (2006). “The reliability coefficient for internal consistency (KR 20) of the OKT (revised 2011) for the total scale was .839. For the Nutrition and Exercise subscales they were .83 and .81, respectively. Validity of the OKT was evaluated by content validity. Questions were examined for difficulty, effectiveness of distracters, and discrimination” (P. Gendler, personal communication, October 27, 2011).

### 2.4. Osteoporosis Health Belief Scale

The OHBS examines the individual's health beliefs about developing osteoporosis. The OHBS has 42 items and consists of two subscales: the Osteoporosis Health Belief Calcium Scale (OHBCS) and the Osteoporosis Health Belief Exercise Scale (OHBES). The OHBCS and OHBES share three subscales: perceptions of osteoporosis regarding seriousness, susceptibility, and general health motivation. The subscales measuring the concepts of barriers and benefits are specific to calcium intake and exercise behavior and are different for the two scales. The participants rate each item using a 5-point scale (1: strongly disagree to 5: strongly agree). The range of scores for each subscale is 6 to 30, with a possible total range of 42 to 210 [15]. The researchers determined internal consistency of the OHBCS and OHBES by Cronbach alpha reliability with coefficients ranging from .61 (health motivation) to .80 (susceptibility). 

“The construct validity of the OHBCS and OHBES was determined by factor analysis. The percentages of variance explained by susceptibility, barriers, benefits, seriousness and heath motivation were 14.4, 12.4, 9.1, 7.7, and 5.8, respectively. The percentages of variance for the OHBES accounted for susceptibility, benefits exercise, barriers exercise, seriousness, and health motivation were 15.9, 12.1, 9.2, 6.4, and 5.7, respectively” [15].

### 2.5. Osteoporosis Self-Efficacy Scale

The OSES is a twenty-one-item rating scale. The OSES tool consists of two subscales: the OSE-Exercise scale and OSE-Calcium scale. The items are rated by the participants on their confidence about engaging in osteoporosis preventive behaviors [17]. The researchers report that the reliability and validity of the OSES were accomplished through use of a principle factor analysis. The OSE-Exercise and OSE-Calcium had internal consistency estimates of .94 and .93, respectively [17].

### 2.6. Educational Intervention

 After completing the OKT, OHBS, and OSES, the participants were presented an educational program entitled *Healthy Bones; Build Them for Life *purchased from the NOF. This education program is a 45-minute Power Point presentation that was developed by the NOF in an effort to increase awareness about bone health and osteoporosis and to promote bone health practices. The participants were also given five handouts developed by the NOF: *Who Gets Osteoporosis: Factors That Put You at Risk*, *Building Bones to Last a Lifetime, What You Should Know about Calcium, Vitamin D and Bone Health, *and *Exercise for Your Bone Health.*

The participants were sent home with a postage paid envelope, void of the participants address, containing the OKT, OHBS, and OESE. They were asked to complete the surveys two months after attending the educational program and return them. A reminder email was sent at the six-week mark. After the surveys were returned, the participants were sent a $20 gift card of their choosing. 

## 3. Results

 Initially the sample included 20 women with a family history of osteoporosis and 22 women without a family history of osteoporosis. The posttest was returned by only 37 of the women, 18 women with a family history of osteoporosis, and 19 women without a family history. This was an 88% return rate. The mean age for women with a family history was 44–46 and 47–49 both at 38.9%. The mean educational level for the women with a family history was 50% with some college/associate degree. All of the women with a family history of osteoporosis reported race as Caucasian. The mean age for women without a family history of osteoporosis was 47–49 and 50–52 both at 32%. The education level for the women without a family history was 36% for both some college/associate degree and baccalaureate degree. Eighty-six percent of the women without a family history of osteoporosis were Caucasian. There was no significant statistical difference between the two groups. This is displayed in Table 1.

### 3.1. Knowledge

Knowledge was analyzed by the total of knowledge questions answered correctly on the Osteoporosis Knowledge Test. To analyze the differences between the two group's knowledge scores, independent *t*-tests were calculated. There was no statistically significant difference in the knowledge of osteoporosis on the pretest between women with a family history of osteoporosis with a mean of 12.8 questions answered correctly and women without a family history of osteoporosis with a mean of 12.2 questions answered correctly. Almost all of the participants in both groups (95%) knew that having a parent or grandparent with osteoporosis increases a person's chance of developing osteoporosis. Additionally, 83% of all participants knew that a diet low in dairy products, being menopausal (88%), smoking daily (81%), and having an eating disorder (88%) increase a person's risk of developing osteoporosis. However, only 16% of all the participants knew that elderly men had an increased chance of developing osteoporosis, and only 4.2% of all the women knew the amount of exercise recommended per week to strengthen bones. While 83% of all of the women knew that vitamin D is required for the absorption of calcium, only 16% knew the daily recommended amount of vitamin D required for the absorption of calcium, and only 12% knew that salmon was a good food source for vitamin D.

To analyze the differences between the two group's knowledge scores after the osteoporosis-specific education intervention, a paired *t*-test was calculated. The results revealed that there was a statistically significant increase in knowledge scores in both groups. For women with a family history the posttest mean was 25.8 questions answered correctly (*t* = −10.214; *df* = 17, *P* < .001). The women without a family history had a highly significant difference in posttest scores with the mean of 26.11 questions answered correctly (*t* = −21.881; *df* = 18; *P* < .001). This is shown in Table 2. 

### 3.2. Health Beliefs

 Health beliefs were examined with the Osteoporosis Health Belief Scale. To analyze health belief differences on the pretest between the women with a family history of osteoporosis and the women without a family history of osteoporosis, independent *t*-tests were calculated. There were a possible range of 42 to 210 for the total OHBS and a possible range of 6 to 30 for the subscales. There was a statistically significant difference with regard to perceived susceptibility to developing osteoporosis between women with a family history of osteoporosis (mean 21.66) and those without a family history of osteoporosis (mean 17.31) (*t* = 3.403; *df* = 38.04; *P* = .032). There was no statistically significant difference in perceived seriousness, benefits of exercise, benefits of calcium intake, barriers to exercise, and barriers to calcium intake between the two groups, nor was there a significant difference observed between the two groups on health motivation. 

 To analyze the differences between the two group's health belief's scores after the osteoporosis-specific education intervention, a paired *t*-test was calculated. The results for the women with a family history of osteoporosis showed a slight increase in mean perceived susceptibility, seriousness, benefits to exercise, benefits to calcium, and health motivation. The only comparison that showed a statistical difference was in the benefits of exercise (*t* = −3.072; *df* = 17; *P* = .007). The women without a family history of osteoporosis had a slight increase in mean perceived susceptibility, benefits of calcium, and health motivation. The only statistically significant difference was in the benefit of calcium (*t* = −5.189; *df* = 18; *P* < .001). This is displayed in Table 3.

### 3.3. Self-Efficacy

When analyzing the results from the two subscales of the Osteoporosis Self-Efficacy Scale, the OSE-Exercise scale, and OSE-Calcium Scale, independent *t*-tests were calculated. On the pretest the women with a family history of osteoporosis had a mean confidence of 60% for self-efficacy to exercise and a 78% confidence in self-efficacy for calcium consumption. The women without a family history had a mean confidence of 62% for self-efficacy to exercise and a 79% confidence in self-efficacy for calcium consumption. 

 In analyzing the differences between the two group's self-efficacy scores after the osteoporosis-specific education intervention, a paired *t*-test was calculated. The mean self-efficacy scores increased for both groups. The women with a family history of osteoporosis had a mean confidence of 62% for self-efficacy to exercise and an 80% confidence in self-efficacy for calcium consumption. The women without a family history of osteoporosis had a mean confidence of 67% for self-efficacy to exercise and an 83% confidence in self-efficacy for calcium consumption. Although the women without a family history of osteoporosis had a higher mean confidence of self-efficacy to exercise and for calcium consumption, there were no significant findings. This is displayed in Table 3.

## 4. Discussion

The aims of this pilot study were to determine if having a family history of osteoporosis impacts knowledge, health beliefs, and self-efficacy regarding osteoporosis among perimenopausal women between the ages of 42 and 52 and secondly to describe the impact an osteoporosis-specific educational intervention had on the knowledge, health beliefs, and self-efficacy for this same population. It was determined that study participants had limited knowledge of osteoporosis and that there was no difference in knowledge between women who have a family history of osteoporosis and women who do not have a family history of osteoporosis. The major finding of this study was that osteoporosis-specific education clearly increases knowledge. Previous work also supports this finding [7, 8]. 

 It was a sensible finding that women with a family history of osteoporosis felt more susceptible to developing osteoporosis than those who do not have a family history of osteoporosis, as women with a family history of osteoporosis have almost double the risk of developing osteoporosis. After the education program, perceived susceptibility did increase in both groups, although this finding was not statistically significant. Previous work with a group of younger women had a consistent finding [7]. The women in both groups perceived the barriers to exercise and calcium as low and the benefits of exercise and calcium as high. This finding is consistent with findings in previous work with younger women and adolescent girls [7, 11]. It is an interesting finding in this study that the women with a family history of osteoporosis perceived exercise more beneficial and women without a family of osteoporosis perceived calcium more beneficial after the education program. This supports further exploration of the health beliefs of perimenopausal women regarding osteoporosis. 

The three criteria of self-efficacy are the conviction that one has the ability to initiate the activity, maintain the activity, and persist in performing the activity in the face of obstacles [17]. It is important to note that in this study self-efficacy total mean scores for both groups of women were above average before the education program. The mean scores for both groups increased after the education program, with the greater impact in the women without a family history of osteoporosis. These findings were not statistically significant, although they are clinically significant.

 The results of the study were limited from the relatively small sample size. The study was also limited because there was limited diversity of the study population. Of the study population, 92% were Caucasian. Each study participant describes their socioeconomic status as middle class. There was an 88% return rate on the second set of surveys.

The major finding of this study was that the osteoporosis-specific education program clearly increased knowledge and some health beliefs in women with and without a family history of osteoporosis. Perimenopausal women are especially vulnerable to bone loss due to the fluctuating and declining levels of estrogen that are needed to maintain bone health. Conversation and education about the risk factors and preventative measures of osteoporosis need to start early in the perimenopausal years and not be delayed to the postmenopausal years as this may reduce morbidity later in life. 

## Figures and Tables

**Table 1:** Demographics.

	Family history		No family history	
	*N*	%	*N*	%
Age				
41–43	1	5.5	3	15.8
44–46	7	38.9	4	21
47–49	7	38.9	6	31.6
50–52	3	16.7	6	31.6
Total	**18**	**100**	**19**	**100**
Years of education				
Some high school (9–11)	3	16.7	2	10.6
High school diploma or GED				
Some college/associate degree	9	50	7	36.8
Baccalaureate degree	4	22.2	7	36.8
Master's degree	2	11.1	3	15.8
Doctoral degree				
Total	**18**	**100**	**19**	**100**
Ethnicity				
White	18	100	16	84.2
Black			2	10.5
Latino			1	5.3
Other				
Total	**18**	**100**	**19**	**100**

**Table 2:** Mean knowledge scores of pre- and posttests.

	Family history		No family history	
	(*n* = 18)		(*n* = 19)	
	Mean		Mean	
	Pre	Post	Pre	Post
Knowledge score	12.66 (SD = 1.57)	25.83 (SD = 5.19)	12.11 (SD = 2.02)	26.11 (SD = 2.67)

*P* < .001.

**Table 3:** Health beliefs and self-efficacy scores.

Health belief	Family history			No family history		
	(*n* = 18)			(*n* = 19)		
	Mean	SD	*P*	Mean	SD	*P*
Susceptibility						
Pre	21.6667	4.61455	.524	17.3158	5.83145	.161
Post	22.5556	5.46887		19.1053	6.07266	
Seriousness						
Pre	16.6667	3.00979	.149	16.2105	3.32631	.702
Post	18.2222	5.13987		15.8421	5.06911	
Benefits-exercise						
Pre	26.3333	2.54374	**.007**	26.0000	2.74874	.630
Post	27.8889	2.42266		26.7368	5.50598	
Benefits-calcium						
Pre	23.3333	3.89570	.197	23.6842	2.05623	**.000**
Post	24.3333	3.53137		26.0000	2.66667	
Barriers-exercise						
Pre	11.4444	3.51839	.570	11.9474	3.56641	.357
Post	11.9444	3.65372		11.3158	4.34681	
Barriers-calcium						
Pre	11.5000	3.65014	.623	12.0526	3.47169	.087
Post	11.8333	3.01467		11.2632	3.72443	
Health motivation						
Pre	21.7778	3.62273	.106	23.6316	3.56231	.220
Post	22.9444	4.70884		24.4737	4.27354	
Self-efficacy exercise						
Pre	60.5500	25.43659	.751	61.3421	26.18002	.377
Post	61.8222	22.31216		67.3526	32.82699	
Self-efficacy calcium						
Pre	77.5859	13.24289	.502	79.2057	17.61927	.313
Post	79.5909	13.63192		83.4067	17.01370	
